# Allelic Variation at the 8q23.3 Colorectal Cancer Risk Locus Functions as a Cis-Acting Regulator of *EIF3H*

**DOI:** 10.1371/journal.pgen.1001126

**Published:** 2010-09-16

**Authors:** Alan M. Pittman, Silvia Naranjo, Sanni E. Jalava, Philip Twiss, Yussanne Ma, Bianca Olver, Amy Lloyd, Jayaram Vijayakrishnan, Mobshra Qureshi, Peter Broderick, Tom van Wezel, Hans Morreau, Sari Tuupanen, Lauri A. Aaltonen, M. Eva Alonso, Miguel Manzanares, Angela Gavilán, Tapio Visakorpi, José Luis Gómez-Skarmeta, Richard S. Houlston

**Affiliations:** 1 Section of Cancer Genetics, Institute of Cancer Research, Sutton, United Kingdom; 2 Centro Andaluz de Biología del Desarrollo, CSIC-UPO, Seville, Spain; 3 Institute of Medical Technology, University of Tampere and Tampere University Hospital, Tampere, Finland; 4 Department of Pathology, Leiden University Medical Center, Leiden, The Netherlands; 5 Department of Medical Genetics, University of Helsinki, Helsinki, Finland; 6 Centro Nacional de Investigaciones Cardiovasculares, Madrid, Spain; 7 Centro Nacional de Investigaciones Biomédica en Red Enfermedades Raras (CIBERER), Universidad Pablo de Olavide-CSIC, Seville, Spain; Stanford University School of Medicine, United States of America

## Abstract

Common genetic variation at human 8q23.3 is significantly associated with colorectal cancer (CRC) risk. To elucidate the basis of this association we compared the frequency of common variants at 8q23.3 in 1,964 CRC cases and 2,081 healthy controls. Reporter gene studies showed that the single nucleotide polymorphism rs16888589 acts as an allele-specific transcriptional repressor. Chromosome conformation capture (3C) analysis demonstrated that the genomic region harboring rs16888589 interacts with the promoter of gene for eukaryotic translation initiation factor 3, subunit H *(EIF3H)*. We show that increased expression of *EIF3H* gene increases CRC growth and invasiveness thereby providing a biological mechanism for the 8q23.3 association. These data provide evidence for a functional basis for the non-coding risk variant rs16888589 at 8q23.3 and provides novel insight into the etiological basis of CRC.

## Author Summary

Common inherited variation on human chromosome 8q23 influences the risk of developing colorectal cancer (CRC). To understand the basis of this association we have compared the frequency of common genetic variants at 8q23 in ∼2,000 CRC cases and ∼2,000 healthy controls. Functional analyses of variants strongly associated with CRC risk showed that the single nucleotide polymorphism rs16888589 underscores the 8q23.3 association. The region of the genome harboring rs16888589 increases the expression of the gene for eukaryotic translation initiation factor 3, subunit H. We show that increased expression of this gene increases CRC growth thereby providing a biological mechanism for the 8q23.3 association. This finding is of particular importance in elucidating the etiological basis of CRC.

## Introduction

Although inherited susceptibility is responsible for ∼30% of all CRC [1], high-penetrance germline mutations in *APC*, the mismatch repair (MMR) genes, *MUTYH*, *SMAD4*, *BMPR1A* (*ALK3*) and *STK11* account for <6% of all CRC [2]. Recent genome-wide association (GWA) studies we have conducted have vindicated a polygenic model of susceptibility to CRC based on the co-inheritance of multiple low-risk variants [3]–[9].

As the SNPs (or markers) genotyped during GWA studies are generally not themselves strong candidates for causality, enumeration of the genetic and functional basis at a specific locus poses a significant challenge. However, as demonstrated by recent studies of the 8q24 and 18q21 risk loci for CRC [10]–[12], dissecting the genetic and functional basis of associations identified by GWA studies can provide novel insights into cancer biology.

We have recently shown that common variation at 8q23 defined by the SNP rs16892766 influences CRC risk [5], [9], [13]. To elucidate a basis of this association we have systematically interrogated the 8q23 association signal through targeted re-sequencing, linkage disequilibrium (LD) mapping and functional analyses.

He we show that a variant mapping to 8q23.3 may influence the transcriptional regulation of eukaryotic translation initiation factor 3, subunit H (*EIF3H*), MIM 603912. These data provides strong support for the functional significance of this SNP and may explain the association observed for CRC at this locus.

## Results

To investigate the 8q23 association and estimate the fraction of common variation at this locus, we generated a fine scale map of a 300 Kb region encompassing the rs16892766 association signal (117,650,000–117,950,000bps) using data from 154 SNPs directly genotyped in 1,964 CRC cases and 2,081 controls and an additional 112 SNPs imputed from HapMap (Dataset S1).

**Figure 1:**
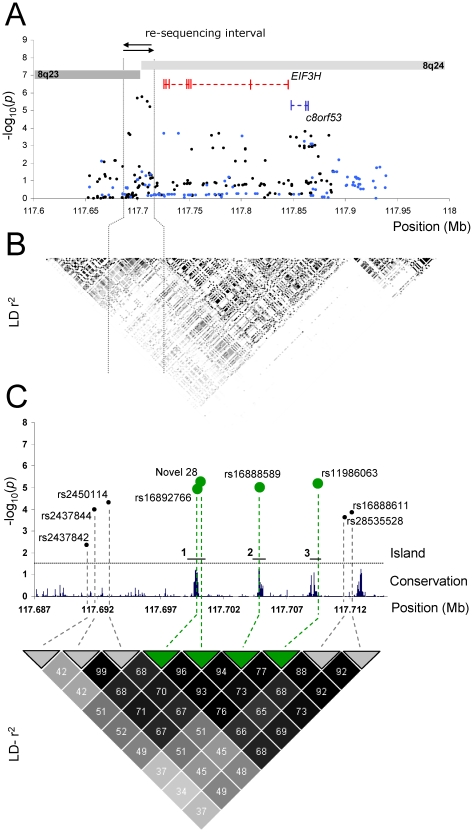
Association between SNPs and colorectal cancer risk at 8q23. (A) Single marker association statistics (-log_10_ *P*) of directly genotyped SNPs (•, black) and imputed SNPs (•, blue). (B) Linkage disequilibrium (LD) statistics (r^2^) of HapMap project data phase II. The darker the shading indicates stronger LD between SNPs. (C) Details of the 22 Kb interval which was resequenced. SNPs displaying the strongest association which were evaluated in biological assays are shown (•, green). Also shown is the sequence conservation across the region in mammals.

A 22 kb genomic region of linkage disequlibrium (LD; Chr8:117,690,773–117,712,909; UCSC March 2006 assembly, NCBI build36.1) capturing rs16892766 provided the best evidence for the 8q23 CRC association signal (Figure 1). To annotate the region we re-sequenced 90 CEU CEPH HapMap individuals (30 trios of U.S. residents of northern and western European ancestry included in the HapMap Phase II project) as this cohort is sufficient to capture all common variation (MAF>5%) [14]. Only 389 bps (1.8%) of the 22 kb region was refractory to re-sequencing owing to low-complexity genomic sequence. We identified 103 variants (Table S1); these included 97 SNPs and six insertion/deletion polymorphisms. Of the 103 variants, 74 were common (minor allele frequency [MAF] ≥0.05) but only 29 had been genotyped by HapMap.

We calculated pair-wise LD statistics between each of the 74 common SNPs and rs16892766. Eight polymorphisms (7 SNPs, 1 insertion/deletion) showed evidence of high LD with rs16892766 (r^2^≥0.50; Figure S1 and Table S1). We genotyped these nine polymorphisms in our series of 1,964 CRC cases and 2,081 controls. The strongest associations were provided by Novel SNP 28 (117,700,195 bp; *P* = 4.55×10^−6^), rs16892766 (*P* = 1.13×10^−5^), rs16888589 (*P* = 8.42×10^−6^) and rs11986063 (*P* = 5.49×10^−6^), (Figure 1; Table S2). To explore the possibility that we may have failed to identify a potential candidate variant we re-examined the association and using data generated from sequencing the 90 CEU CEPH HapMap individuals imputed all untyped SNPs in the case-control cohort and tested them for association with CRC (Figure S2). No additional SNP to the nine directly genotyped, provided superior evidence for an association (*P*>1.16×10^−4^) indicating our selection of candidate SNPs based upon our LD criteria was sufficient to identify a candidate variant. Analysis of the four most significantly associated SNPs-rs16892766, Novel 28, rs16888589 and rs11986063 showed they are strongly correlated with one another (*i.e.* pairwise r^2^>0.75; Figure 1) and constitute a single risk haplotype (*P*>8.61×10^−6^). Collectively these data support the hypothesis that one of these four variants is likely to contribute to the 8q23 CRC association.

The promoter of the nearest protein-coding gene, *EIF3H* (eukaryotic translation initiation factor 3, subunit H), maps 140 kb telomeric to rs16892766; hence it is possible that rs16892766 or another SNP in LD, affects an unknown element controlling *EIF3H* expression. We excluded the possibility the 8q23 association signal is a consequence of a long-range LD with a coding sequence change in *EIF3H* by resequencing the transcribed regions and splice site boundaries of *EIF3H*. Only synonymous SNPs were identified and none of these showed evidence of correlation with rs16892766 (*i.e.* r^2^<0.01, *D'*<0.01)

Sequence conservation in non-coding regions has been shown to be a good predictor of cis-regulatory sequences [15]. Moreover, it has been proposed that variation with evolutionary-conserved regions is likely to be associated with phenotypic differences that may contribute to expression of traits [15]. Cross-species sequence comparison of the 22 kb interval revealed the presence of three islands conserved between *mammals* annotated by rs16892766/Novel 28, rs16888589 and rs11986063 (Figure 1). To further examine the nature of the sequence within the 22 kB region we implemented a number of computational methodologies. Using ESPERR (evolutionary and sequence pattern extraction through reduced representations), which searches for potential regulatory sequences, all three islands are predicted to have regulatory potential [16]. Using Enhancer Element Locator (EEL) software [17] the strongest EEL-predicted regulatory element mapped to island 1 as indicated in Figure 1.

**Figure 2:**
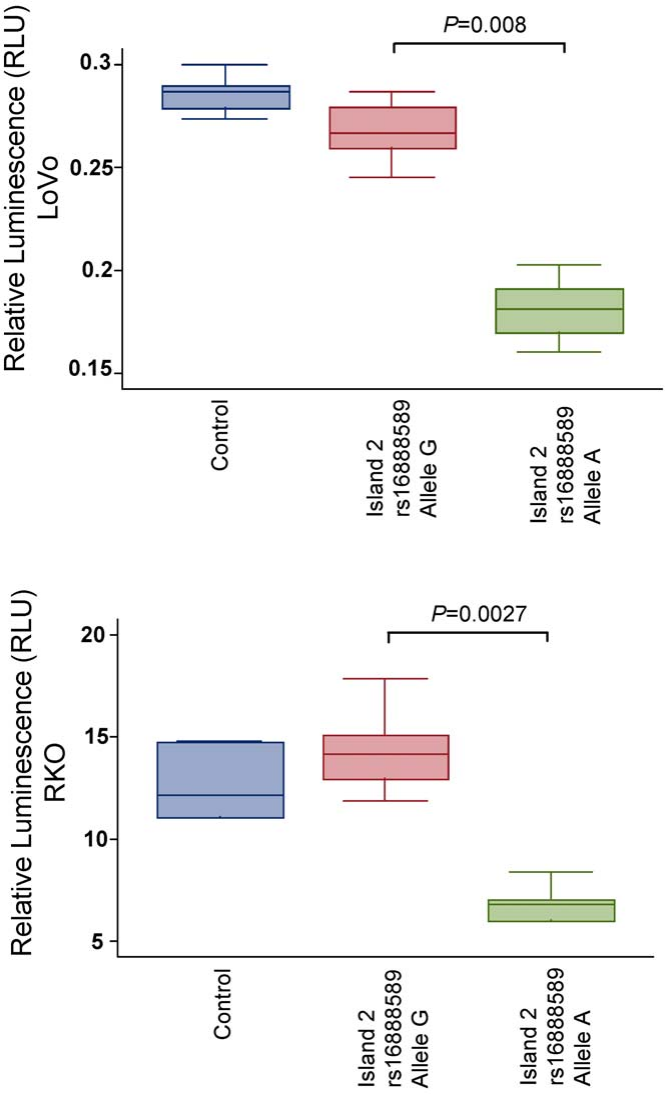
Reporter gene activity for the island 2 construct incorporating rs16888589. Luciferase reporter assays demonstrating repressor activity in LoVo and RKO CRC cell lines.

To evaluate the potential enhancer activity of the three putative regulatory regions, we cloned DNA fragments containing the three conserved islands, incorporating the different alleles of rs16892766-Novel 28, rs16888589 and rs11986063, into GFP or *LacZ* reporter vectors designed to assay enhancer activity in zebrafish, *Xenopus* and mice transgenic assays [10], [18], [19] or into *Luc2* reporter vectors to evaluate regulatory activity in human CRC cell lines. Although no enhancer activity was detected for any island in the different transgenic experiments, the cell culture assays were compatible with island 1 and 3 acting as weak enhancers. However, no allele-specific differences were observed for the polymorphisms mapping to these islands (Figure S3). In contrast, luciferase assays demonstrated that island 2 functions as a repressor that was allele-specific (Figure 2). The ancestral A allele, but not the risk G allele of rs16888589, significantly repressed *luc2* reporter gene expression (*P*<0.01; Figure 2). These data are consistent with island 2 in the CRC risk region natively functioning as a repressor in allele-specific manner.

**Figure 3:**
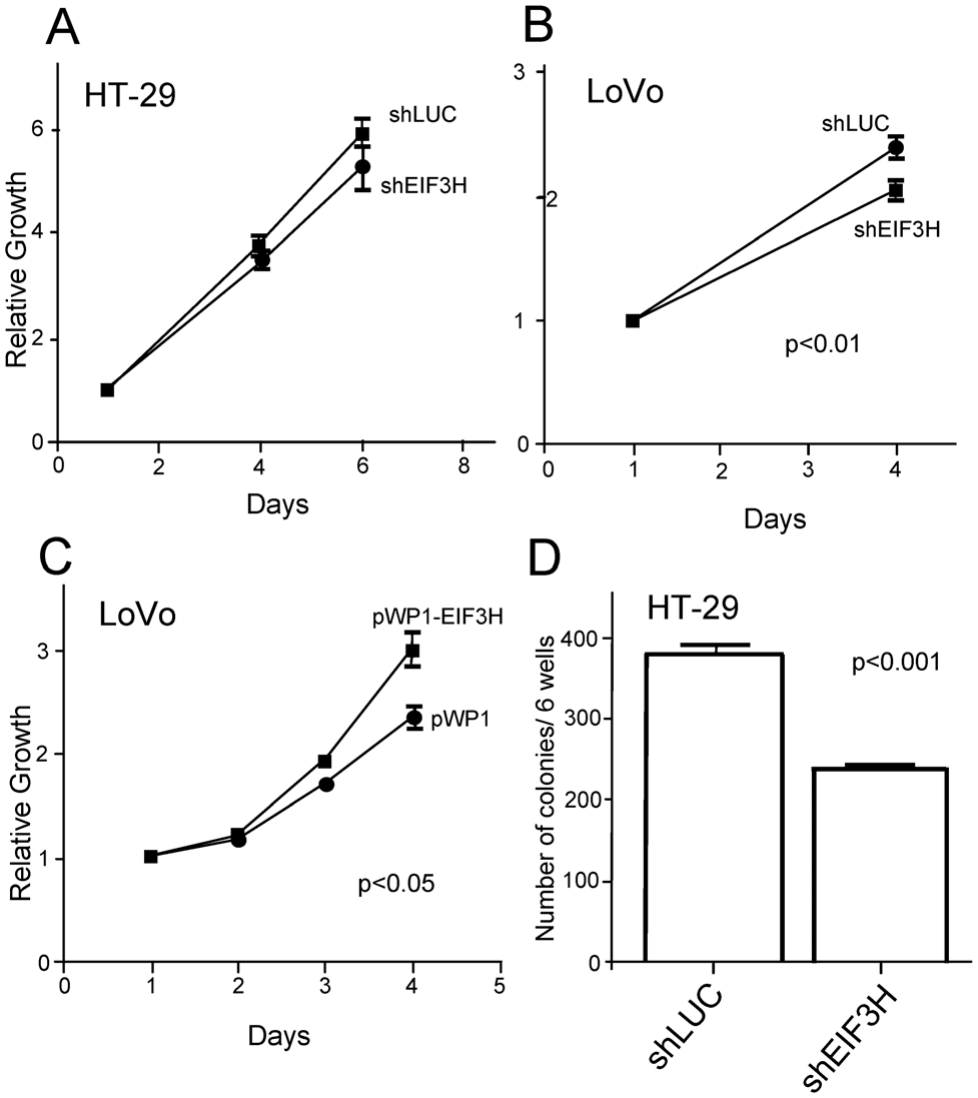
Impact of differential *EIF3H* expression on growth of colorectal cancer cell lines. CRC cell line growth based on AlamarBlue analysis. (A) HT-29 cells transduced with shRNA against EIF3H (shEIF3H) and control (shLUC). No effect on cell growth is seen. (B) LoVo cells transduced with shRNA against EIF3H (shEIF3H) and control (shLUC). Suppression of EIF3H reduced the cell growth of LoVo. (C) LoVo cells transduced with lentivirus carrying EIF3H expression vector (pWP1-EIF3H) and empty vector control (pWP1). The EIF3H overexpression increased significantly the cell growth of LoVo. Mean values ±SEM are shown. (D) Impact of EIF3H expression on anchorage-independent growth of HT-29 colorectal cancer lines in soft agar assay. HT-29 cells transduced with pLL3.7 lentivirus carrying shRNA against EIF3H (shEIF3H) or against luciferase (shLUC) were grown in soft agar followed by counting the colonies. Approximately 40% reduction in the number of colonies was found. Mean values ±SEM are shown.

To investigate the effect of differential eIF3H expression on the malignant phenotype of CRC; we selected LoVo, which has two copies of *EIF3H*; and HT-29 which has high gene amplification and expression of *EIF3H* (Figure S4). In the LoVo CRC line, reduction of *EIF3H* levels by short interfering RNA (shRNA) reduced cell proliferation (Figure 3). Conversely, *EIF3H* up-regulation by transfection with lentivirus carrying an EIF3H expression vector (pWP1-EIF3H) increased cellular proliferation (Figure 3). In the CRC cell line HT-29 we were unable to achieve complete knock-down of *EIF3H*, (Figure S4) most likely due to the high basal level of expression present in this cell line. However, anchorage-independent growth measured by soft agar assay was associated with a 40% reduction in the number of colonies with *EIF3H* knock-down (Figure 3). Collectively these findings provide evidence that high eIF3h levels influence the establishment and maintenance of CRC.

We have previously found no association between *EIF3H* expression in EBV-transformed lymphoblastoid cells and 8q23 risk genotype [5]. Likewise, we found no association between rs16892766 and *EIF3H* mRNA expression in a series of colorectal adenomas and carcinomas (Figure S5), or absence of copynumber gain of 8q23 and *EIF3H* genotype.

Hypothesising that the 22 Kb region of 8q23 physically interacts with the *EIF3H* we used chromosome conformation capture (3C) to examine for interaction with the *EIF3H* promoter. We interrogated a constant promoter fragment against a series of fragments in LoVo and RKO CRC cell lines and a control fibroblast cell line (Figure 5). In both CRC cell lines we observed strong interaction between a fragment encompassing the promoter and island 2 but not island 1 or island 3. Thus, the rs16888589 risk region physically interacts with *EIF3H*. Intriguingly, a STAT binding site (TTC**C**GGGAA) with differential allele affinity by rs16888589 is predicted by TFsearch. Direct evidence for support for allele specific functional consequences for rs16888589 were provided by electrophoretic mobility shift assays (EMSA) showing greater affinity for nuclear protein-DNA complex formation with the risk allele (*P* = 5.3×10^−4^; Figure 5).

**Figure 4:**
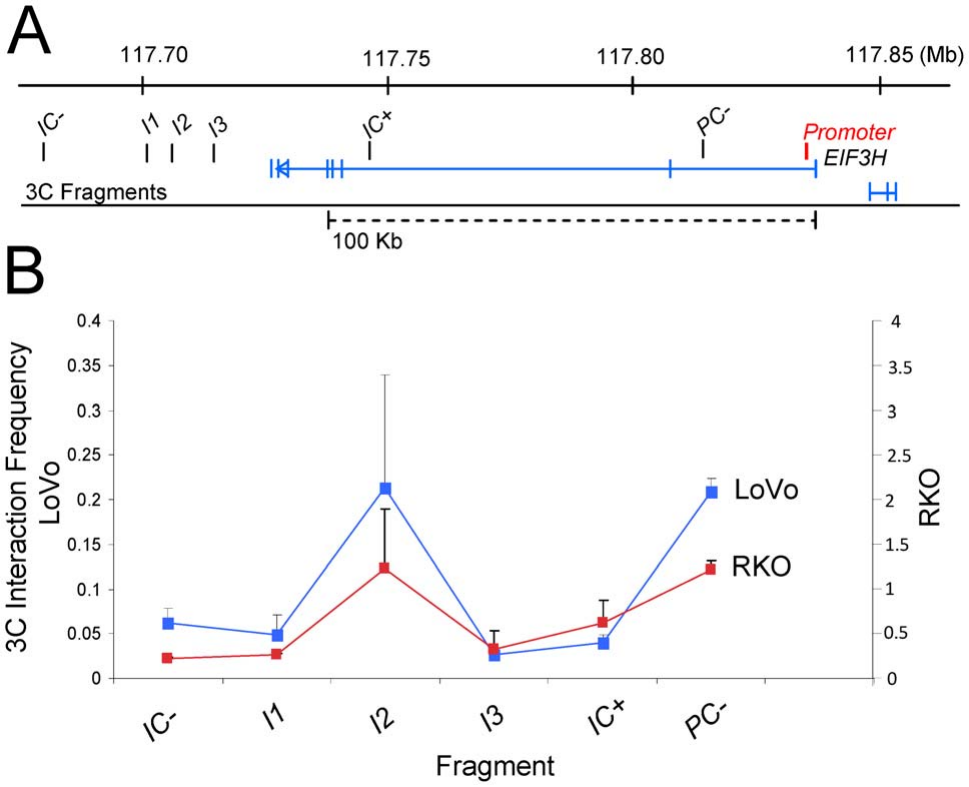
Physical interaction of the CRC variant rs16888589 with *EIF3H* in CRC cell lines. (A) Physical map of the region interrogated by 3C. The position of the constant fragment at the *EIF3H* promoter is shown in red. Genomic positions of target fragments (Table S3) are denoted by black bars. (b) Graph showing the 3C interaction frequency containing the promoter with each target fragment in the LoVo and RKO CRC cell lines. Error bars represent SEM. The results demonstrate increased interaction frequency in the cell lines between the *EIF3H* promoter and fragment I2 to which rs16888589 maps. The Y-axis refers to interaction frequency; RKO to the right; LoVo to the left. The assay was performed independently for two and three times in RKO and LoVo colorectal cancer cell lines, respectively. Labels at each data point in the graph denote the name of each target fragment.

We have recently shown that the G allele of the 8q24 variant rs6983267 is preferentially amplified during development of CRC [20]. To examine whether a similar phenomenon exists for the 8q23 locus, we analyzed allelic imbalance at rs16892766. Analysis of the 220 heterozygous tumors revealed copy number change in 20% (44/220) of the CRCs. rs6983267 is located 10.8 Mb distally from rs16892766 and it is known that amplified regions in 8q can be large [20]. Therefore, the G allele specific amplification of the rs6983267 containing region could have an impact on the allelic imbalance data on rs16892766. Thus, we restricted the allelic imbalance analysis to the 133 rs6983267 homozygous tumors. Of 133 tumors, 27 showed allelic imbalance. In 11 tumors the risk allele C was preferred whereas in 16 tumors the A allele was favoured. No significant difference in the selection of the targeted alleles was observed (*P* = 0.44).

## Discussion

In this work we have performed functional analysis and 3C studies using CRC cell lines to maximize detection of a subtle functional effect associated with rs16888589. Using these model systems we were able to demonstrate that possession of the A allele of rs16888589 may have repressor function on *EIF3H*. This does not exclude the possibility of the regulatory region we identified influences other genes through cis- and trans-effects. In addition, we cannot rule out the possibility that other, rarer risk alleles within the LD block may also contribute to the risk of CRC.

eIF3H is one of the 13 putative subunits of the eukaryotic translation initiation factor 3 (EIF3). At the cellular level, *EIF3H* overexpression increases proliferation, growth and survival. eIF3h appears to function through translation, as the initial appearance of overexpressed eIF3h in rapidly induced NIH-3T3 cells correlates tightly with the stimulation of protein synthesis and the generation of malignant phenotypes. Overexpression of *EIF3H* is seen in prostate, breast, and liver cancer and overexpression of eIF3h malignantly transforms immortal NIH-3T3 cells [21]–[23]. High level amplification of the *EIF3H* has also been associated with advanced stage and poor prognosis prostate cancer [24]. This is a general feature of eIF3h, as high levels also affect translation, proliferation, and a number of malignant phenotypes of CHO-K1 and HeLa cells and, most significantly, of primary prostate cell line [23].

Reduction of eIF3h levels in breast and prostate cancer cell lines by shRNA methods has previously been shown to reduce cell proliferation and anchorage-independent growth in soft agar [22]. In our study we have now shown that that manipulating eIF3h expression has a similar effect in CRC. These data provide compelling evidence that high eIF3h levels directly stimulate protein synthesis, resulting in the establishment and maintenance of the malignant phenotype of CRC.

We found no association between rs16892766 and *EIF3H* mRNA expression in a series of colorectal adenomas and carcinomas. This is perhaps not entirely surprising given the moderate effect of the variant on enhancer activity and the relatively small numbers of samples analysed. Additionally, CRC occurs late in life and it is likely that only a cumulative long-term imbalance in *EIF3H* expression will influence CRC development. Finally, expression differences may only be relevant to a specific subpopulation of cells such as intestinal stem cells.

It has recently been suggested that analysis of transcript abundance provides a means of establishing a relationship between genotype an expression [25]. Hence analysis of the impact of rs16888589 genotype and transcript abundance in different cell lineages of colonic tissue at different stages of development and may prove highly informative.

Overexpression of eIF3h has recently been shown to inhibit Myc-dependent induction of apoptosis of primary prostate cells and *EIF3H* and *MYC* and may cooperate in enhanced protein translation either in a general way or for a specific subset of mRNAs [23]. We have previously shown that the G allele of the 8q24 variant rs6983267 is preferentially amplified during development of CRC [20]. In contrast there appears to be no such selection for 8q23 alleles according to rs16892766 genotype.

In summary, we identified rs16888589 as a genetic risk variant for CRC at 8q23.3 and i*n vitro* experiments showed a functional significance of this SNP. We propose that this risk allele of rs16888589 acts as part of a *cis*-regulatory element for the *EIF3H* promoter in CRC, which may mediate CRC risk through control of *EIF3H* expression.

## Materials and Methods

### Ethics

Ethical committee approval for this study was obtained from relevant study centres (UK, MREC02/0/97, Netherlands, LUMC/CME P04.124 and the University of Helsinki).

### Resequencing-SNP discovery panel

DNA was extracted from 30 CEPH mother-father-child trios (n = 90; U.S. Utah residents with northern and western Europe ancestry; Coriell cell depositories); Phase I and II HapMap cohort.

### Genotyping cohort

1,964 CRC cases (964 male; mean age at diagnosis 58 years; SD, 8) ascertained through The National Study of Colorectal Cancer Genetics (NSCCG) [26]. 2,081 healthy individuals (845 males; mean age 57 years; SD, 9) were recruited from NSCCG (871), Genetic Lung Cancer Predisposition Study (1999–2004; n = 706) [27], and the Royal Marsden Hospital Trust/Institute of Cancer Research Family History and DNA Registry (1999–2004; n = 504). All cases and controls were UK residents and had self-reported European ancestry.

### Resequencing

Sequence changes in 8q23 (117,690,773–117,712,909; UCSC March 2006 assembly, NCBIbuild36.1) were identified by sequencing. PCR and sequencing primers were designed using Primer3 software (sequences available on request). Amplicons were sequenced by ABI chemistry (BigDye v3.1; Applied Biosystems, Foster City, US) and implemented on ABI 3730xl DNA analyzers (Applied Biosystems, Foster City, US). Sequence reads were analyzed using Mutation Surveyor software v3.10 (Softgenetics, State College, US). For QC purposes all chromatograms were visually inspected for base independently by two researchers.

### Genotyping

DNA was extracted using conventional methodologies and quantified using PicoGreen (Invitrogen, Renfrew, UK). Custom genotyping was conducted using the Illumina Golden Gate system (Illumina Inc, San Diego, US) or by Kaspar (Kbiosciences, Hertfordshire, UK). Assay details available on request. Genotyping quality control was tested using duplicate DNA samples. For all SNPs, >99.9% concordant results were obtained.

### Enhancer reporter assays in Xenopus, zebrafish and mice

The allele-specific fragments (Table S3) of each Island were PCR-amplified, sub-cloned into PCR8/GW/TOPO vector and verified by PCR and direct sequencing. Gateway technology was then used to transfer the DNA fragment to the corresponding destination reporters. For zebrafish transgenesis, we transferred the DNA fragments to the ZED destination vector [18]. This vector contains the *Xenopus* Cardiac actin promoter driving DsRed as a positive control for transgenesis. Zebrafish transgenic embryos were generated as described [18]. Three or more independent stable transgenic lines were generated for each construct. *Xenopus laevis* transgenic embryos were generated using the I-SceI method [28] with the reporter vector recently described [10]. For the generation of transgenic mice, the genomic fragments were transferred into a vector containing the human minimal beta-globin promoter, *lacZ* and a SV40 polyadenylation signal [19]. Afterward, vectors were linearized, the vector backbone removed and the construct microinjected into one cell mouse embryos. F0 embryos of 11, 5–13 dpc stages were harvested and stained for *lacZ* activity.

### Luciferase assay

The allele-specific fragments of each Island were transferred from PCR8/GW/TOPO vectors into pGL3 *luc2* using the gateway technology. pGL3 *luc2* constructs were amplified in *E.coli* followed by purification of plasmid DNA using Qiagen Endotoxin-free Maxi-prep kits. LoVo (Human colon adenocarcinoma) and RKO CRC cell lines (ECACC, Salisbury, UK) were grown in F12 (Ham's) and McCoy's 5a culture medium, respectively, supplemented with 10% FCS (37°C, 100% relative humidity, 5% CO_2_). Cultured cells were seeded in 96-well tissue culture (Greiner) plates, 2.7×10^5^ cells/well, in 200 µl of media and grown for ∼24 hours until 80% confluent. Transient transfection was carried out with Transfast transfection reagent (Promega, Southampton, UK) at a charge ratio of 1∶1 of transfection reagent to DNA in serum free medium. In each well, cells were transfected with 150 ng of pGL3-construct DNA and 5 ng of the internal control plasmid DNA (pRL-CMV, Promega) that encodes the *Renilla* luciferase gene under the control of the CMV promoter. Six replicates of cells, both LoVo and RKO were transfected by each reporter construct. Each transfection experiment was repeated twice. Transiently transfected cells were grown for 48 hours, following which the luciferase assay was carried out using the Dual-Glo luciferase assay system (Promega, Southampton, UK) as per the manufactures instructions. Firefly luciferase (from the pGL3 constructs) and *renilla* luciferase (from the pRL-CMV internal control) were measured sequentially on a 96-well (Dynex Inc, West Sussex, UK). The ratio of luminescence from the experimental reporter to the luminescence from the control reporter was calculated for each sample, defined as the relative luciferase activity. Difference in relative activity of each experiment was assessed using the Mann-Witney test.

### Chromosome conformation capture assay (3C)

3C assay was performed as previously described [29]. Adherent cultured LoVo or RKO cells were processed to get a single cell preparation. 10^7^ cells were fixated with 2%PFA, lysated, and nuclei were digested with *Hind*III (Roche, West Sussex, UK). DNA was then ligated with T4 DNA ligase (Promega, Southhampton, UK) in low concentration conditions to favour intramolecular ligations. A set of locus specific primers (Table S3) were designed close to the *Hind*III site. The primer near to *EIF3H* promoter acting as the fixed primer, and different interactions were tested using primers close to each island. Two negative control primers were mapping 30 kb upstream and 20 kb downstream the three islands (Figure 4; Table S3). PCR products were run in an agarose gel and quantified using a Typhoon scanner. Product values were normalised to a control composed of a BAC containing all test fragments. We validated the ligation product of Island 2 (I2) with the promoter fragment of *EIF3H* (promoter) by sequencing the band from the agarose gel.

**Figure 5:**
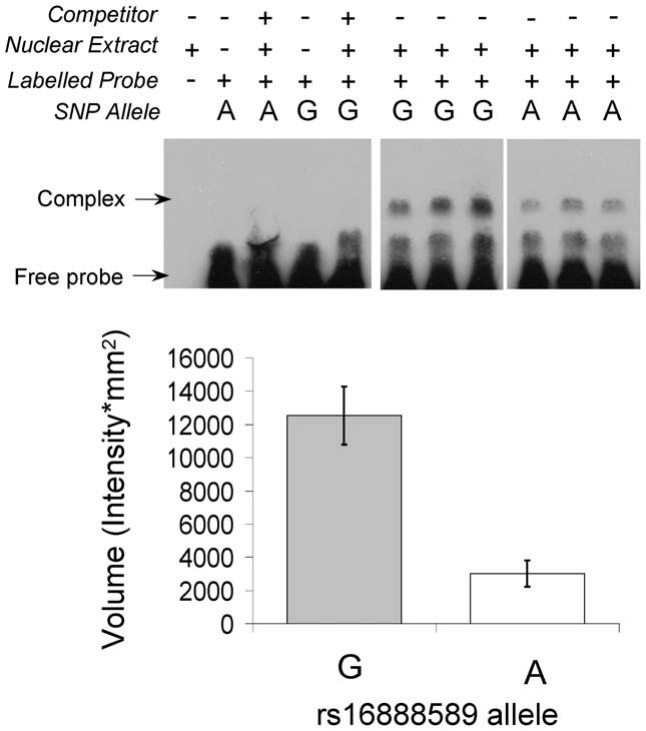
Electro-mobility shift assay (EMSA) of rs16888589 showing differential binding of nuclear protein for A and G alleles. Upper panel shows autoradiographs for binding of double stranded A-allele and G-allele probes to lymphoblastoid nuclear extracts; lower panel shows a 4-fold difference in binding between A and G alleles.

### Electrophoretic mobility shift assay (EMSA)

Biotin end-labeled and unlabeled complementary oligonucleotide probes (5′-CCTTCTCTCTTCCC**A**GAACCCGGCTGTCCC-3′–Biotin and 5′- CCTTCTCTCTTCCC**G**GAACCCGGCTGTCCC-3′) (Invitrogen, Renfrew, UK) were annealed to generate double-stranded EMSA probes. Nuclear protein was extracted from a lymphoblastoid cell line using NE-PER nuclear and cytoplasmic extraction kits (Thermo Scientific, Loughborough, UK).

EMSA experiments were performed using the Lightshift Chemiluminescent EMSA Kit (Pierce, Thermo Scientific, Loughborough, UK). Each 20 ul binding reaction contained 20 fmols of biotin end-labeled target DNA, 10× binding buffer, 50 ng Poly(dI.dC), 2.5% glycerol, 0.05% NP-40 and 5 ug of nuclear protein extract. After a 20 minute incubation, reactions were electrophoresed for 1 h at 100 V in a 6% polyacrylamide gel (0.5% TBE buffer) and then electroblotted for 1 h at 30 V. Chemiluminescent detection of biotin end-labeled DNA was performed with a strepdavidin-horseradish peroxidase conjugate captured onto X-ray film and developed according to the manufacturers instructions. Omitting nuclear extract and addition unlabelled probes (1000-fold excess) served as controls. Quantification of intensity signals was performed using a BioRad CCD Molecular Imager FLUOR-S (Biorad, Hemel Hampstead, UK).

### EIF3H over-expression and knockdown in colorectal cancer cell lines

#### Cell lines

HT-29 and LoVo CRC cell lines were obtained from DSMZ (Braunschweig, Germany), and cultured under recommended conditions.

#### Fluorescence in situ hybridization

Dual-color fluorescence *in situ* hybridization (FISH) with digoxigenin-dUTP (Roche Diagnostics, West Sussex, UK) labeled PAC probe for *EIF3H* and FITC-dUTP (NEN, Boston, MA, USA) labeled pericentromeric probe for chromosome 8 (pJM128) were hybridized to interphase nuclei of the cell lines as previously [30]. After stringent washes, slides were stained with antidigoxigenin-rhodamine (Roche Diagnostics) and counterstained with an antifade solution (Vectashied, Vector Laboratories, Burlingame, CA, USA) containing 4,6-diamidino-2-phenylindole (DAPI). FISH signals were scored using Olympus BX50 epifluorescence microscope (Olympus Inc, Tokyo, Japan).

#### Lentivirus production and transductions

Lentiviral constructs were performed as previously described [21]. The sequences of short hairpin RNAs (shRNAs) are given in Table S3. shRNA oligos were obtained from Sigma-Proligo (The Woodlands, TX, USA) and cloned into the lentiviral plasmid Lentilox3.7 (pLL3.7). cDNA clone of *EIF3S* was obtained from Geneservice (Cambridge, UK) and cloned into the lentiviral plasmid WPI. Both pLL3.7 and pWPI –plasmids contain green fluorescence protein (GFP) as a reporter gene. Cells that showed >90% transduction efficiency were used for the experiments. To enhance the viral transduction, 8 µg/ml of polybrene (Sigma-Aldrich, Milwaukee, WI, USA) was used in every transduction.

#### Quantitative real-time RT-PCR (q-RT-PCR)

The expression levels of *EIF3H*, and a housekeeping gene TATA-box binding protein (*TBP*) were analyzed using previously described methodology [21] Briefly, PCR reactions were performed using the LightCycler apparatus (Roche Diagnostics, Mannheim, Germany) with the LC Fast Start DNA SYBR Green I Kit (Roche Diagnostics). Melting curve analysis and agarose gel runs were performed to ensure the formation of specific PCR products.

#### Growth curves and soft agar assays

Growth curves and soft agar assays were performed as previously described [21]. For the growth curves cells transduced with lentiviruses were plated on a 24-well plate at 50 000 cells/ml density. Alamar Blue (Trek Diagnostic Systems, Cleveland, OH, USA) was added to the wells followed by fluorescence measurement after two hours of incubation. Values were normalized against day 1. Each experiment was performed in five replicates and repeated at least twice. For soft agar assay, cells transduced with lentiviruses were trypsinized and dilution of 5000 cells/well was mixed with 5% agarose to form 0.35% upper layer. After two weeks, colonies were photographed under UV-microscope and counted. All experiments were conducted in triplicate and repeated at least twice.

### Allelic analysis imbalance analysis

Allelic imbalance in the CRC tumors was scored as described in Tuupanen et al. [20].

### EIF3H expression and 8q23 copy number analysis

Snap-frozen rectal adenomas and carcinomas from patients who had not received radiotherapy or adjuvant chemotherapy were evaluated for *EIF3H* expression and 8q copy number. Frozen tumors were macrodissected in a cryostat to achieve tumor percentage assessed (50–80%), guided by H&E-sections. DNA was isolated from tumors using the Genomic Wizard kit (Promega, Madison, WI). Copy numbers were analyzed using GeneChip Mapping 10 K 2.0 arrays (Affymetrix, Inc., Santa Clara, CA) as described previously [31]. RNA was isolated from tumours using the Qiagen RNeasy mini kit with DNaseI digestion (Qiagen Sciences, Germantown, MD) and quality checked by lab-on-a-chip (Agilent Technologies, Agilent Technologies, Palo Alto, California). 2 ug RNA was hybridized to human 35 K oligo microarrays from the CMF of the Netherlands Cancer Institute as previously described [32] Comparison of the difference in expression levels was assessed using the Mann-Whitney test.

### Statistical and bioinformatics analysis

Statistical analyses were undertaken in Stata v10 (Station College, US). Deviation of the genotype frequencies in the controls from those expected under Hardy-Weinberg Equilibrium (HWE) was assessed by χ^2^ test. Unconditional logistic regression was used to calculate the per allele odds ratio (OR) of CRC and associated 95% confidence intervals (CIs) for each SNP. Haplotype analysis was conducted in Haploview software (v4.0) and tested for association via a likelihood ratio test. Linkage disequilibrium metrics were calculated using Haploview software (v4.0).The weight of evidence in favour of each associated SNP was quantified by calculating Akaike weights [33]. Prediction of the untyped SNPs in the case-control data were performed with MACH1.0 on reference phased haplotypes from HapMap phase II data (January 2007 on NCBI B35 assembly, dbSNP build 125) and the SNP-discovery panel. Reference haplotypes were constructed of all SNPs identified in the re-sequenced interval by use of PHASE software [34].

## Supporting Information

Dataset S1 Association Results of SNPs genotyped by Illumina Golden Gate and by Imputation with MACH 1.0.
(0.04 MB XLS)

Figure S1 LD plot of SNPs (MAF≥0.05) identified through re-sequencing of the 22 Kb interval. Short-listed SNPs highlighted in turquoise are correlated with rs16892766 (r2 LD≥0.5).
(0.13 MB PDF)

Figure S2 Single marker association statistics (-log10P) of custom genotyped short-listed SNPs (Green) and remaining SNPs (Blue) that were imputed in our case-control cohort using phased haplotypes from the CEPH SNP discovery panel as our reference. Also plotted are individual quality scores for each imputed SNP.
(0.02 MB PDF)

Figure S3 Luciferase reporter assays of genomic Islands 1, 2 and 3 in LoVo cell lines.
(0.02 MB PDF)

Figure S4 Copy number and expression analysis of EIF3H in LoVo and HT-29.
(0.06 MB PDF)

Figure S5 EIF3H expression in 36 rectal adenomas and 43 carcinomas. Histological subtype split by rs16892766 genotype
(0.02 MB PDF)

Table S1 SNPs identified from re-sequencing the 22 Kb interval (Figure 1).
(0.17 MB DOC)

Table S2 Association of candidate SNPs with risk of CRC.
(0.04 MB DOC)

Table S3 Primers and shRNA sequences used.
(0.04 MB DOC)
