# Supplementary material for: Allelic Variation at the 8q23.3 Colorectal Cancer Risk Locus Functions as a Cis-Acting Regulator of EIF3H
Source: PLoS Genet. 2010 Sep 16;6(9):e1001126. doi: 10.1371/journal.pgen.1001126 (PMC2940760; doi:10.1371/journal.pgen.1001126)

Figure S1. LD plot of SNPs (MAF ≥ 0.05) identified through re-sequencing of the 22 Kb interval. Short-listed SNPs highlighted in turquoise are correlated with rs16892766 ($r2$ LD ≥ 0.5).

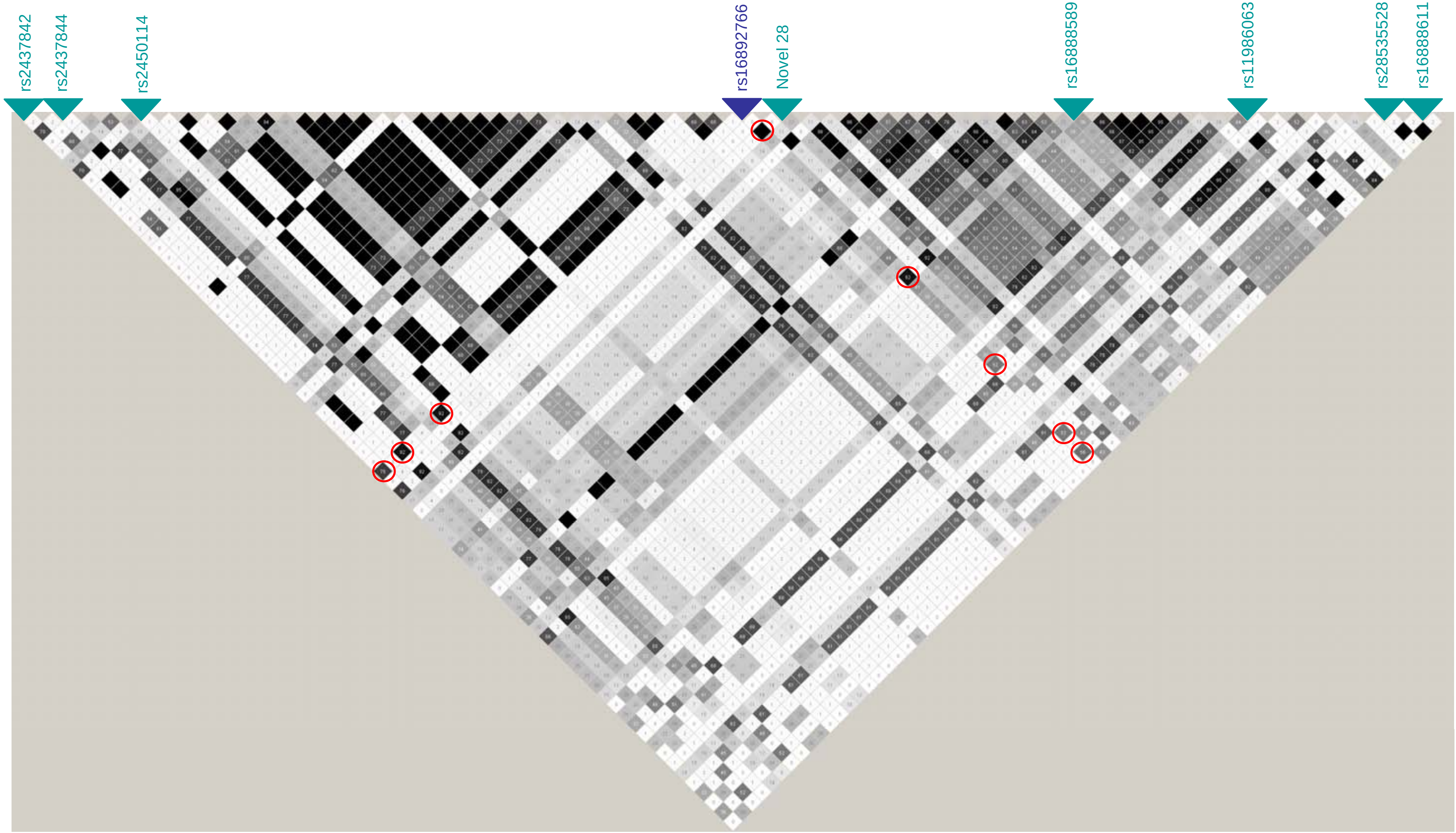

Supplement: Figure S1 — LD plot of SNPs (MAF≥0.05) identified through re-sequencing of the 22 Kb interval. Short-listed SNPs highlighted in turquoise are correlated with rs16892766 (r2 LD≥0.5). (0.13 MB PDF) [file pgen.1001126.s002.pdf]
