# Supplementary material for: Allelic Variation at the 8q23.3 Colorectal Cancer Risk Locus Functions as a Cis-Acting Regulator of EIF3H
Source: PLoS Genet. 2010 Sep 16;6(9):e1001126. doi: 10.1371/journal.pgen.1001126 (PMC2940760; doi:10.1371/journal.pgen.1001126)
Supplement: Figure S2 — Single marker association statistics (-log10P) of custom genotyped short-listed SNPs (Green) and remaining SNPs (Blue) that were imputed in our case-control cohort using phased haplotypes from the CEPH SNP discovery panel as our reference. Also plotted are individual quality scores for each imputed SNP. (0.02 MB PDF) [file pgen.1001126.s003.pdf]

Figure S2. Association of IMPUTED SNPs in the 22kb interval

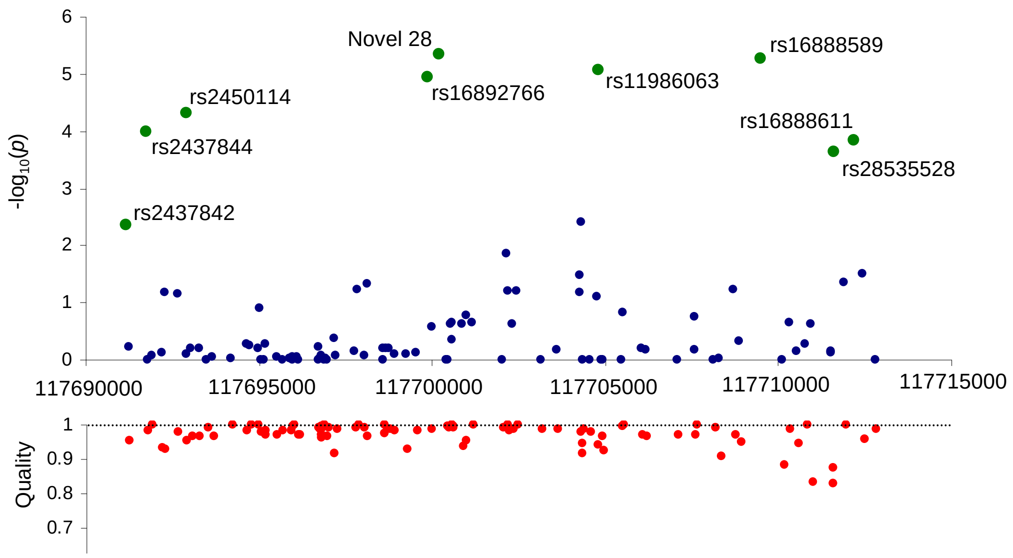

Panel shows single marker association statistics (-log10*P*) of custom genotyped short-listed SNPs (Green) and remaining SNPs (Blue) that were Imputed in our case-control cohort using phased haplotypes from the CEPH SNP discovery panel as our reference. Also plotted are individual quality scores for each imputed SNP.
