# Supplementary figures and images for: Allelic Variation at the 8q23.3 Colorectal Cancer Risk Locus Functions as a Cis-Acting Regulator of EIF3H
Source: PLoS Genet. 2010 Sep 16;6(9):e1001126. doi: 10.1371/journal.pgen.1001126 (PMC2940760; doi:10.1371/journal.pgen.1001126)

**Figure S3.** Luciferase reporter assays of genomic Islands 1, 2 and 3 in LoVo cell lines.

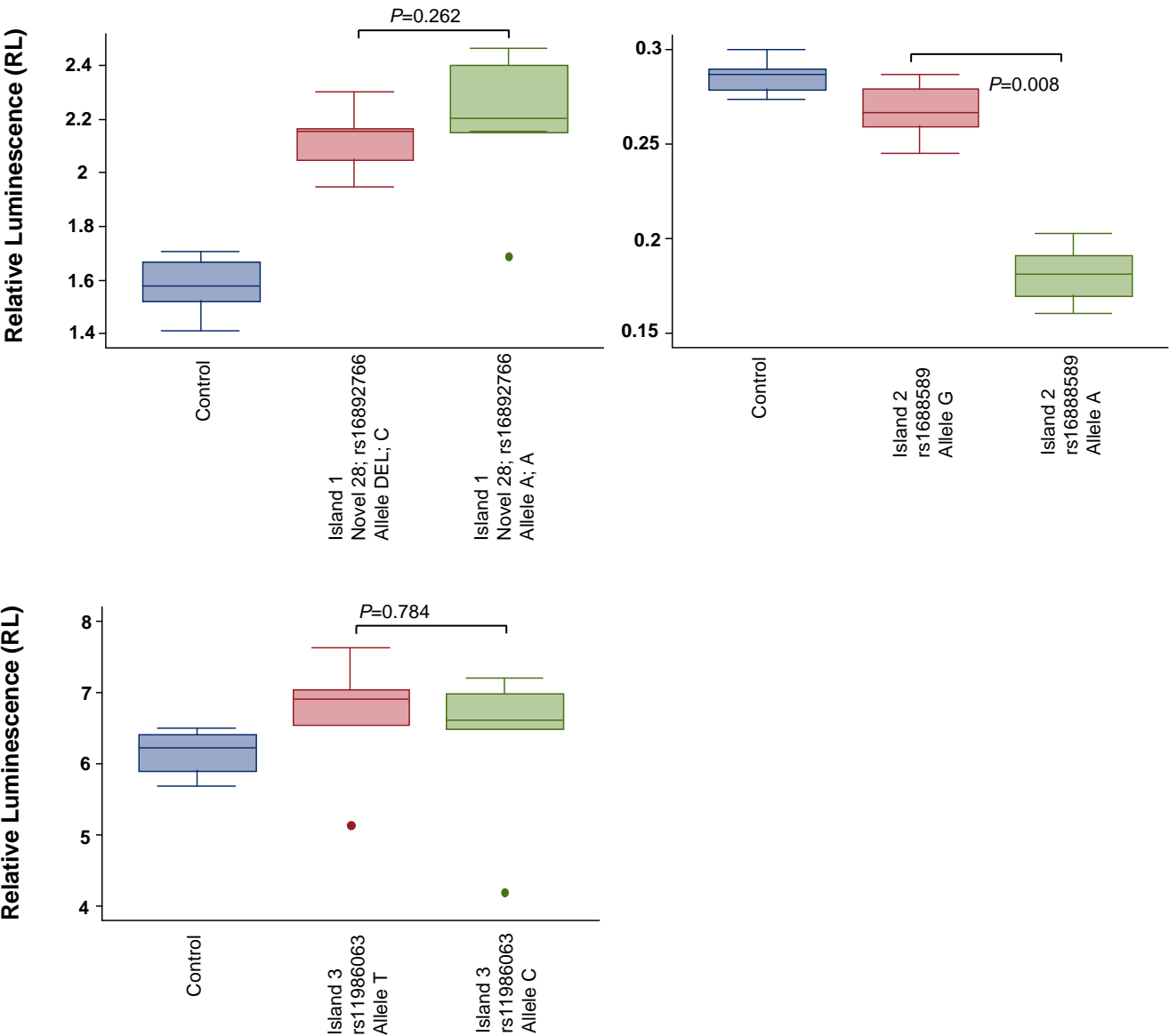

Supplement: Figure S3 — Luciferase reporter assays of genomic Islands 1, 2 and 3 in LoVo cell lines. (0.02 MB PDF) [file pgen.1001126.s004.pdf]
