# Supplementary material for: Allelic Variation at the 8q23.3 Colorectal Cancer Risk Locus Functions as a Cis-Acting Regulator of EIF3H
Source: PLoS Genet. 2010 Sep 16;6(9):e1001126. doi: 10.1371/journal.pgen.1001126 (PMC2940760; doi:10.1371/journal.pgen.1001126)
Supplement: Figure S4 — Copy number and expression analysis of EIF3H in LoVo and HT-29. (0.06 MB PDF) [file pgen.1001126.s005.pdf]

Figure S4. Copy number and expression analysis of *EIF3H* in LoVo and HT-29

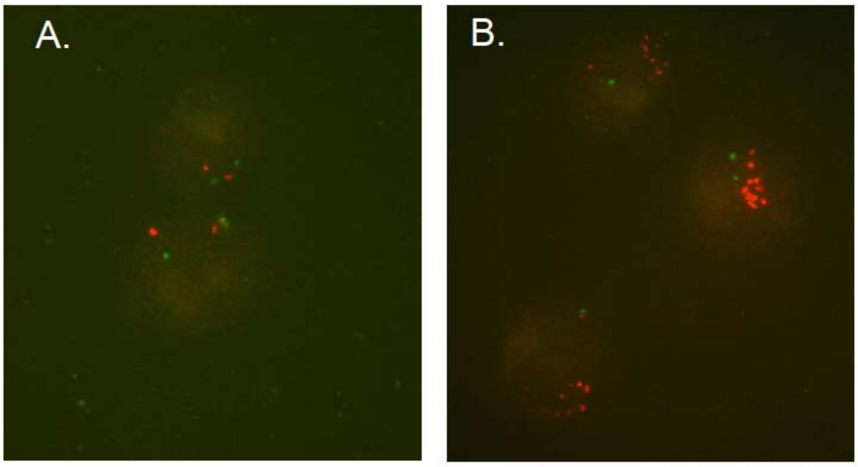

FISH analysis of *EIF3H* copy number in CRC cell lines (A) LoVo and (B) H-29. Multiple red signals show high-level amplification of *EIF3H* in HT-29, whereas only two copies of the gene were demonstrated in LoVo. Green signals correspond to the two copies of chromosome 8 centromeres in both cell lines.

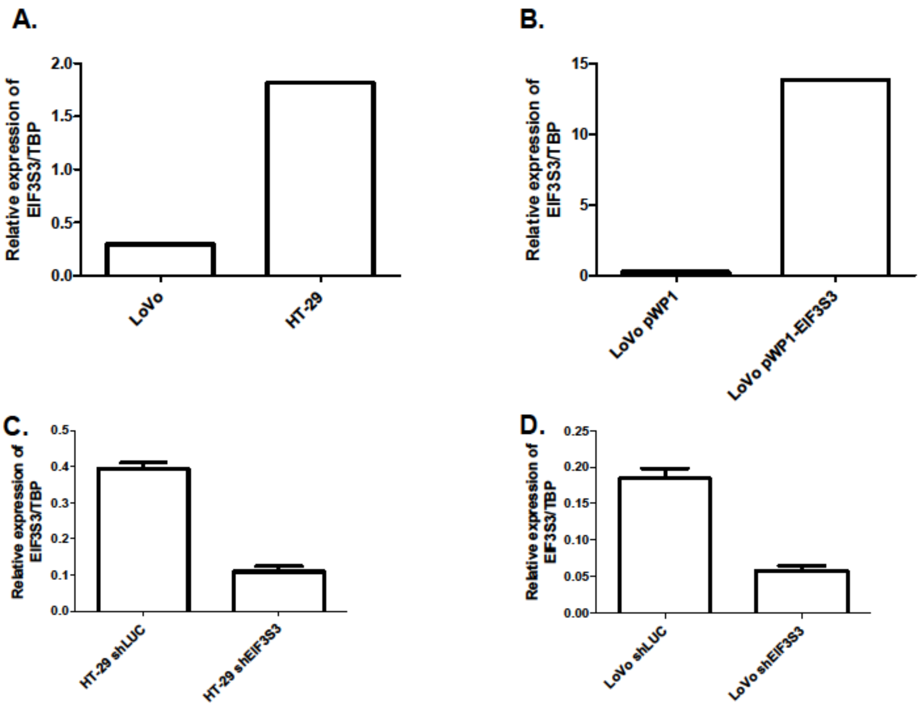

Expression analysis of *EIFH* by Q-RT-PCR. (A) Relative expression of *EIF3H* in LoVo and HT-29 cell lines. (B) Transduction with lentivirus carrying pWP1-EIF3H leads to >20-fold overexpression of *EIF3H* in LoVo, C) silencing of EIF3H with shRNA (pLL3.7-EIF3H) in HT-29, and D) LoVo cell lines. Mean values ±SEM are shown.
