# Supplementary material for: Allelic Variation at the 8q23.3 Colorectal Cancer Risk Locus Functions as a Cis-Acting Regulator of EIF3H
Source: PLoS Genet. 2010 Sep 16;6(9):e1001126. doi: 10.1371/journal.pgen.1001126 (PMC2940760; doi:10.1371/journal.pgen.1001126)
Supplement: Figure S5 — EIF3H expression in 36 rectal adenomas and 43 carcinomas. Histological subtype split by rs16892766 genotype (0.02 MB PDF) [file pgen.1001126.s006.pdf]

Figure S5. *EIF3H* expression in 36 rectal adenomas and 43 carcinomas

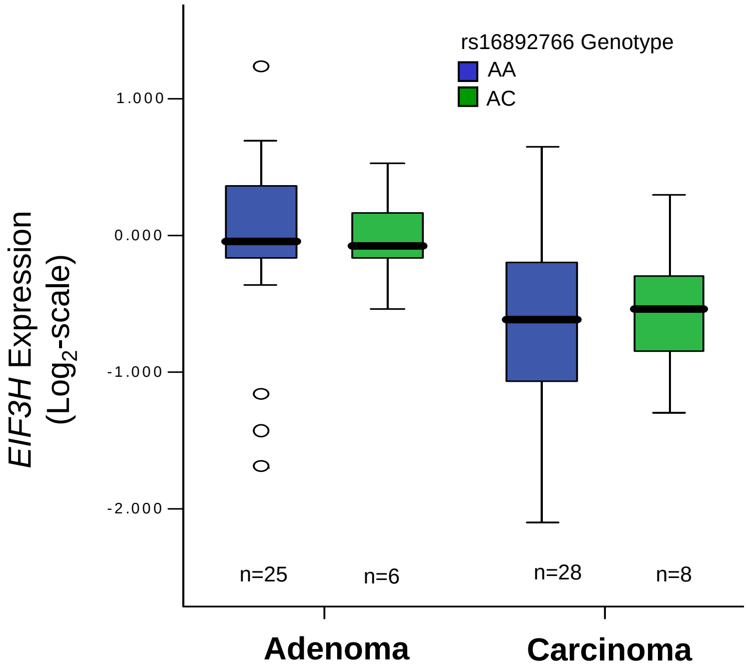

EIF3H expression in 36 rectal adenomas and 43 carcinomas. Histological subtype split by rs16892766 genotype

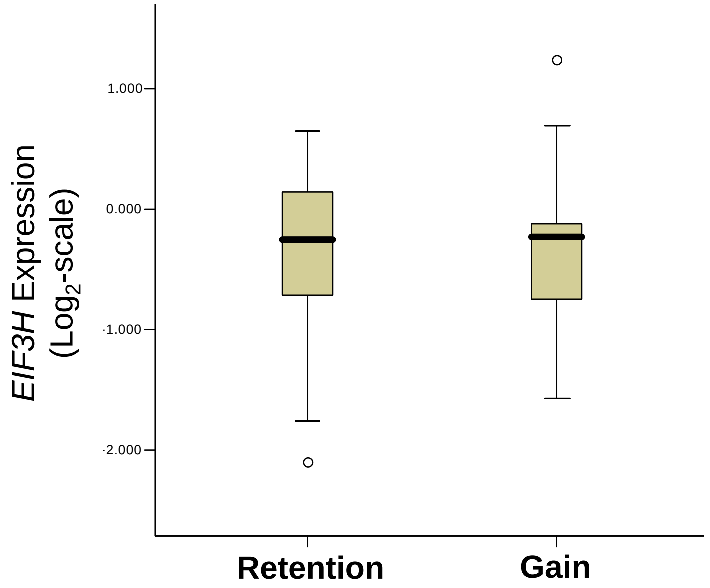

EIF3H expression in 36 rectal adenomas and 43 carcinomas. Split by 8q24 copy number status; Retention and (2) Gain (>2).
