# Supplementary material for: Allelic Variation at the 8q23.3 Colorectal Cancer Risk Locus Functions as a Cis-Acting Regulator of EIF3H
Source: PLoS Genet. 2010 Sep 16;6(9):e1001126. doi: 10.1371/journal.pgen.1001126 (PMC2940760; doi:10.1371/journal.pgen.1001126)
Supplement: Table S1 — SNPs identified from re-sequencing the 22 Kb interval (Figure 1). (0.17 MB DOC) [file pgen.1001126.s007.doc]

**Table S1.** SNPs identified from re-sequencing the 22 Kb interval (Figure 1).

| **Alleles** | **dbSNP ID** | **Genomic Position (bp)** | **pHWE** | **Call Rate (%)** | **MAF**  **(%)** |
| --- | --- | --- | --- | --- | --- |
| C/A | rs2437842 | 117691176 | 1.00 | 100 | 0.09 |
| G/T | rs2437843 | 117691249 | 0.82 | 97.8 | 0.16 |
| G/A | rs2437844 | 117691752 | 1.00 | 100 | 0.13 |
| C/T | Novel 1 | 117691763 | 1.00 | 100 | 0.01 |
| G/C | rs11987719 | 117691895 | 1.00 | 100 | 0.08 |
| T/C | rs2511654 | 117692184 | 0.27 | 100 | 0.23 |
| A/G | rs9650066 | 117692264 | 1.00 | 100 | 0.36 |
| C/T | Novel 2 | 117692660 | 1.00 | 100 | 0.01 |
| G/A | rs2450114 | 117692900 | 1.00 | 100 | 0.13 |
| G/A | Novel 3 | 117692904 | 1.00 | 100 | 0.08 |
| T/C | rs2511655 | 117693039 | 0.78 | 100 | 0.15 |
| T/C | rs2450115 | 117693274 | 0.80 | 98.9 | 0.16 |
| C/T | Novel 4 | 117693492 | 1.00 | 98.9 | 0.00 |
| G/T | rs1529943 | 117693664 | 1.00 | 97.8 | 0.08 |
| T/A | rs11994283 | 117694208 | 1.00 | 100 | 0.08 |
| C/T | Novel 5 | 117694643 | 1.00 | 91 | 0.01 |
| A/G | rs2118107 | 117694742 | 0.56 | 100 | 0.22 |
| T/A | rs2511649 | 117694965 | 0.20 | 92.1 | 0.23 |
| C/G | Novel 6 | 117695026 | 1.00 | 100 | 0.01 |
| A/G | Novel 7 | 117695064 | 1.00 | 100 | 0.01 |
| A/G | rs1968495 | 117695158 | 1.00 | 100 | 0.08 |
| A/G | Novel 8 | 117695175 | 1.00 | 100 | 0.01 |
| G/T | rs1529944 | 117695509 | 1.00 | 100 | 0.08 |
| C/T | Novel 9 | 117695669 | 1.00 | 100 | 0.01 |
| G/C | rs7003608 | 117695906 | 1.00 | 100 | 0.08 |
| G/T | Novel 10 | 117695960 | 1.00 | 100 | 0.01 |
| A/G | rs7003645 | 117695980 | 1.00 | 100 | 0.08 |
| A/G | rs7007682 | 117696114 | 1.00 | 100 | 0.08 |
| A/G | Novel 11 | 117696137 | 1.00 | 100 | 0.03 |
| G/T | Novel 12 | 117696704 | 1.00 | 100 | 0.01 |
| G/T | rs2437845 | 117696730 | 0.78 | 100 | 0.15 |
| A/C | Novel 13 | 117696763 | 1.00 | 100 | 0.08 |
| C/T | Novel 14 | 117696792 | 1.00 | 100 | 0.08 |
| C/G | Novel 15 | 117696794 | 1.00 | 100 | 0.08 |
| A/C | Novel 16 | 117696795 | 1.00 | 100 | 0.08 |
| A/T | Novel 17 | 117696796 | 1.00 | 100 | 0.08 |
| C/T | rs4415350 | 117696863 | 1.00 | 100 | 0.00 |
| A/C | Novel 18 | 117696934 | 1.00 | 100 | 0.08 |
| A/G | Novel 19 | 117696981 | 1.00 | 100 | 0.01 |
| C/T | Novel 20 | 117697166 | 1.00 | 95.5 | 0.06 |
| C/T | Novel 21 | 117697223 | 1.00 | 95.5 | 0.07 |
| C/T | Novel 22 | 117697748 | 1.00 | 96.6 | 0.01 |
| A/G | rs7842524 | 117697856 | 1.00 | 100 | 0.36 |
| C/T | Novel 23 | 117698037 | 1.00 | 100 | 0.08 |
| C/T | rs4876669 | 117698109 | 1.00 | 100 | 0.36 |
| -/G | Novel 24 | 117698589 | 1.00 | 100 | 0.01 |
| G/C | rs3133285 | 117698592 | 0.78 | 100 | 0.15 |
| G/T | rs3133286 | 117698661 | 0.78 | 100 | 0.15 |
| C/T | rs3102738 | 117698758 | 0.78 | 100 | 0.15 |
| -/AAGA | Novel 25 | 117698898 | 1.00 | 100 | 0.08 |
| G/C | Novel 26 | 117699260 | 1.00 | 93.3 | 0.09 |
| G/A | Novel 27 | 117699532 | 1.00 | 95.5 | 0.08 |
| A/C | rs16892766 | 117699864 | 0.82 | 98.9 | 0.12 |
| C/A | rs17743323 | 117699976 | 1.00 | 100 | 0.06 |
| -/A | Novel 28 | 117700195 | 0.84 | 100 | 0.12 |
| T/C | Novel 29 | 117700409 | 1.00 | 100 | 0.01 |
| T/C | Novel 30 | 117700468 | 1.00 | 100 | 0.01 |
| G/A | rs10105966 | 117700533 | 0.21 | 100 | 0.36 |
| C/T | rs7011668 | 117700563 | 1.00 | 100 | 0.06 |
| G/T | Novel 31 | 117700589 | 1.00 | 100 | 0.01 |
| G/A | rs7822969 | 117700857 | 0.38 | 100 | 0.37 |
| G/A | rs7823142 | 117700969 | 0.21 | 100 | 0.36 |
| G/A | rs6469653 | 117701145 | 0.21 | 100 | 0.36 |
| T/A | Novel 32 | 117702017 | 1.00 | 98.9 | 0.01 |
| C/G | rs6469654 | 117702146 | 0.26 | 100 | 0.23 |
| -/T | rs35436018 | 117702185 | 0.07 | 100 | 0.31 |
| C/T | rs12547061 | 117702296 | 0.21 | 98.9 | 0.36 |
| G/A | rs12547129 | 117702438 | 0.07 | 97.8 | 0.31 |
| C/G | Novel 33 | 117703133 | 1.00 | 94.4 | 0.01 |
| C/T | Novel 34 | 117703606 | 1.00 | 98.9 | 0.08 |
| C/T | rs2016144 | 117704250 | 0.08 | 96.6 | 0.30 |
| C/T | rs3887097 | 117704277 | 0.08 | 96.6 | 0.30 |
| C/T | rs5003050 | 117704304 | 0.07 | 95.5 | 0.22 |
| C/T | Novel 35 | 117704331 | 1.00 | 91 | 0.02 |
| C/T | Novel 36 | 117704563 | 1.00 | 98.9 | 0.01 |
| T/C | rs1074584 | 117704758 | 0.18 | 100 | 0.28 |
| A/G | rs16888589 | 117704783 | 0.95 | 100 | 0.11 |
| C/A | Novel 37 | 117704885 | 1.00 | 100 | 0.02 |
| G/A | Novel 38 | 117704913 | 1.00 | 100 | 0.04 |
| G/C | Novel 39 | 117705456 | 1.00 | 98.9 | 0.01 |
| G/A | rs4876363 | 117705510 | 0.38 | 98.9 | 0.26 |
| C/T | rs1094824 | 117706044 | 0.24 | 98.9 | 0.23 |
| T/C | rs7014790 | 117706166 | 0.18 | 97.8 | 0.24 |
| G/A | Novel 40 | 117707067 | 1.00 | 98.9 | 0.02 |
| T/C | rs10955784 | 117707563 | 0.20 | 100 | 0.24 |
| A/C | rs10955785 | 117707584 | 0.04 | 100 | 0.23 |
| C/A | Novel 41 | 117708137 | 1.00 | 100 | 0.01 |
| -/C | rs36006355 | 117708293 | 0.37 | 98.9 | 0.32 |
| T/C | rs2015069 | 117708713 | 1.00 | 100 | 0.05 |
| CT | rs1811409 | 117708880 | 0.15 | 100 | 0.25 |
| C/T | rs11986063 | 117709496 | 0.72 | 100 | 0.13 |
| G/A | rs1198705 | 117710120 | 1.00 | 97.8 | 0.09 |
| C/T | Novel 42 | 117710298 | 1.00 | 97.8 | 0.02 |
| T/C | rs4876365 | 117710519 | 0.15 | 100 | 0.25 |
| A/G | rs11987235 | 117710791 | 1.00 | 95.5 | 0.14 |
| C/T | Novel 43 | 117710935 | 1.00 | 93.3 | 0.09 |
| C/T | rs12542425 | 117711526 | 0.08 | 98.9 | 0.34 |
| G/A | rs12056883 | 117711534 | 0.81 | 98.9 | 0.10 |
| C/T | rs28535528 | 117711609 | 0.71 | 98.9 | 0.13 |
| C/G | rs1370095 | 117711897 | 0.82 | 87.6 | 0.13 |
| C/T | rs16888611 | 117712171 | 0.81 | 95.5 | 0.12 |
| -/T | rs35119067 | 117712424 | 0.90 | 94.4 | 0.12 |
| C/T | Novel 44 | 117712782 | 1.00 | 92.1 | 0.00 |
