# Supplementary material for: Allelic Variation at the 8q23.3 Colorectal Cancer Risk Locus Functions as a Cis-Acting Regulator of EIF3H
Source: PLoS Genet. 2010 Sep 16;6(9):e1001126. doi: 10.1371/journal.pgen.1001126 (PMC2940760; doi:10.1371/journal.pgen.1001126)
Supplement: Table S2 — Association of candidate SNPs with risk of CRC. (0.04 MB DOC) [file pgen.1001126.s008.doc]

**Table S2:** Association of candidate SNPs with risk of CRC

| **SNP ID** | **Genomic**  **Position (bp)** | **MAF**  **Cases**  **(%)** | **MAF**  **Controls**  **(%)** | ***P*_allele_** | **OR_allele_ (95% CI)** | **Log-likelihood** | **Akaike**  **Weight** |
| --- | --- | --- | --- | --- | --- | --- | --- |
| rs2437842 | 117691176 | 0.050 | 0.037 | 4.45E-03 | 0.73 (0.59 to 0.91) | -2798.16 | 25.02 |
| rs2437844 | 117691752 | 0.108 | 0.082 | 1.04E-04 | 0.74 (0.64 to 0.87) | -2794.69 | 4.41 |
| rs2450114 | 117692900 | 0.109 | 0.083 | 4.87E-05 | 0.73 (0.63 to 0.86) | -2794.1 | 3.29 |
| rs16892766 | 117699864 | 0.085 | 0.059 | 1.13E-05 | 0.68 (0.57 to 0.81) | -2792.54 | 1.51 |
| Novel 28 | 117700195 | 0.086 | 0.060 | 4.55E-06 | 0.67 (0.57 to 0.80) | -2791.72 | 1.00 |
| rs16888589 | 117704783 | 0.084 | 0.058 | 8.42E-06 | 0.68 (0.57 to 0.81) | -2792.28 | 1.32 |
| rs11986063 | 117709496 | 0.095 | 0.067 | 5.49E-06 | 0.69 (0.59 to 0.81) | -2792.79 | 1.71 |
| rs28535528 | 117711609 | 0.091 | 0.069 | 2.32E-04 | 0.74 (0.63 to 0.87) | -2795.35 | 6.15 |
| rs16888611 | 117712171 | 0.092 | 0.069 | 1.43E-04 | 0.73 (0.62 to 0.86) | -2794.87 | 4.84 |

Characteristics and association of the nine variants short listed as candidate causal variants with risk of colorectal cancer. MAF, minor allele frequency; OR, odds ratio; CI, 95% confidence interval. Chromosome positions are derived from NCBI build 36.1.
