# Supplementary material for: Allelic Variation at the 8q23.3 Colorectal Cancer Risk Locus Functions as a Cis-Acting Regulator of EIF3H
Source: PLoS Genet. 2010 Sep 16;6(9):e1001126. doi: 10.1371/journal.pgen.1001126 (PMC2940760; doi:10.1371/journal.pgen.1001126)
Supplement: Table S3 — Primers and shRNA sequences used. (0.04 MB DOC) [file pgen.1001126.s009.doc]

**Table S3:** Primers and shRNA used

Primers used to generate luciferase assay constructs:

| **Island** | **Forward primer (5’-3’)** | **Reverse primer (5’-3’)** |
| --- | --- | --- |
| 1 | GCATAGGTGACCGTCTCC | AACTTGGAGGCAGAGAGC |
| 2 | CACACACATACACTTCTCTCCCA | GGCAGCAAAATCTTTAATGAA |
| 3 | TTGGAAACCCTTTTCTGCCAATCA | AGTTCTGCATGATGAAGCGTTTA |

Primers used in 3C analysis:

| **Name** | **ID** | **Primer sequence** |
| --- | --- | --- |
| 3C-EIF3H-IslC- | IC- | CTAAAGAATCCCTGCTTGG |
| 3C-EIF3H-Isl1 | I1 | CAATATTCATTCTCTCTCTCAGG |
| 3C-EIF3H-Isl2 | I2 | GGGTTTATCTGCACTGAGG |
| 3C-EIF3H-Isl3 | I3 | AACAAGGGCTCTATGATTACAC |
| 3C-EIF3H-IslC+ | IC+ | TTCACAAGGGCTCACTTAAC |
| 3C-EIF3H-PromC- | PC- | CCTAAAGGGAAAGATTTAACAG |
| 3C-EIF3H-Prom | Promoter (Reference Probe) | CCTTCTCACAGGAGATTGG |
| 3C-EIF3H-PromC+ | PC+ | ACCTGTTTCATAGGGTCTCC |

The shRNA sequences used:

| **Name** | **Sequence: (5’-3’ direction)** |
| --- | --- |
| shEIF3H | tgcaagtgcagatagatggcttcaagagagccatctatctgcacttgcttttttcACGTTCACGTCTATCTACCGAAGTTCTCTCGGTAGATAGACGTGAACGAAAAAAGAGCT |
| shLUC | TGATTTCGAGTCGTCTTAATTTCAAGAGAATTAAGACGACTCGAAATCTTTTTTCACTAAAGCTCAGCAGAATTAAAGTTCTCTTAATTCTGCTGAGCTTTAGAAAAAAGAGCT |
